# Computers, meaning, and consciousness

**DOI:** 10.1093/nc/niaf057

**Published:** 2025-12-11

**Authors:** Robert Worden

**Affiliations:** Active Inference Institute, 751 2nd St #82, Crescent City, CA, United States

**Keywords:** information in books and computers, encoding and decoding, computation as an interpretation of a physical process, indeterminacy, representational entity, computational functionalism, theories of consciousness, spatial binding, analogue models of space

## Abstract

This paper uses simple arguments to derive a negative conclusion: that a computer cannot be conscious. If the brain is only a neural computer, brains cannot be conscious. Consciousness implies that there is something else happening in the brain, besides computation. In a running computer, information about outside events is encoded, to enable physical computation. The information required to decode the information (e.g. to interpret volts as bits, or neuron spike trains as numbers) is not inside the computer. The meaning of any computation is not defined inside the computer; it is only defined by some external entity which decodes the results. Without decoding information, a computer contains no information about outside events. In the same way, the meaning of any book is not defined inside the book; the book requires outside knowledge to read it. Consciousness contains meaningful information about external events. If the brain is only a computer, without decoding (which requires external information) it contains no information about external events. If the brain is only a computer, consciousness cannot be realised by events inside the brain. This conclusion is compared with philosophical positions on computational functionalism, representation, and intentionality. Something more than neural computing must be happening in the brain. One suggestion is that the something could be an analogue model of 3-D space. An analogue model contains information which requires little or no decoding. Hence, an analogue model of reality in the brain might be the source of consciousness. This merits further investigation.

## Introduction

This paper uses simple arguments to derive a negative conclusion: that computers are not conscious. If the brain is as a neural computer (and only a computer), brains cannot be conscious. To account for consciousness, there must be something else happening in the brain. If this negative conclusion is correct, it has far-reaching consequences for the study of computers, brains, and consciousness.

The conclusion follows from a simple analysis, along the following lines: all information in a running computer is physically encoded, to enable computation. To have meaning, that information requires decoding. The information needed for decoding resides outside the computer. Without decoding, the information physically inside the computer is not about any defined thing; so the computer cannot be aware of any defined things.

Consider the parallel case of a book. Information inside a book consists of printed ink shapes; without decoding, those shapes have no meaning. The information required for decoding the book (such as knowledge of a language) exists only outside the book.

Information inside a book or a computer has no defined meaning, without decoding. This is described in sections 2–5 of the paper. It agrees with conclusions about the nature of computing in (Horsman et al. 2014, Stepney and Kendon 2021). They show that computation depends on an external ‘representational entity’ which interprets the physical events inside the computer as being a computation. For Horsman et al., computation is not defined as a physical process; it is an interpretation (a decoding) of a physical process. The result also agrees with the No-Go theorem for AI computers of (Kleiner and Ludwig 2024).

There are important consequences for theories of consciousness. In this paper, I use the term ‘consciousness’ to refer to phenomenal consciousness, as in (Block 1995), which can also be described as ‘subjective experience’. A person, animal, or system has a conscious experience when there is ‘something it is like’ for the system to be the subject of that experience (Nagel 1974). Phenomenal consciousness is distinguished from access consciousness (Block 1995, 2002). I also assume that consciousness can be about some external thing; it is possible to be conscious of something, as in being conscious of a tree.

It is a reasonable assumption (assumed in most theories of consciousness) that consciousness is realised by physical events in the brain. If the brain is only a computer, information in it is encoded as neurons firing. Without decoding, this information in the brain is not about external things; it might be about anything, or nothing. The information needed for decoding is not inside the brain (it is in the scientific community). Without decoding, physical events in the brain cannot be the origin of consciousness about external things.

The empirical fact of our consciousness implies that the brain is not just a neural computer; to cause consciousness of things, something else must be happening inside brains. That is the second conclusion, described in sections 6 and 7 of the paper. It contradicts most current theories of consciousness.

Most theories of phenomenal consciousness, such as (Tononi 2012, Lamme 2020, Lau 2022), assume that consciousness arises from computational processes in the brain. In this, they follow the assumption of computational sufficiency (Chalmers 2011) or equivalently of computational functionalism (Butlin et al. 2023). The conclusion of this paper is not consistent with computational functionalism. Without decoding, information in the brain is not information about external things, so it cannot support phenomenal consciousness of things. The conclusion implies that many current theories of consciousness cannot be correct as they stand.

These negative conclusions are important, because they bear on the puzzle of phenomenal consciousness, which is one of the greatest challenges in science. The topic of consciousness—in brains and in man-made computers—continues to be of great interest to many researchers. If a negative conclusion can help us know what is not possible, and so clear the ground for new ideas, it will be an important contribution.

The topics of sections 2–7 have been the subject of extensive philosophical debate. Section 8 discusses some leading philosophical contributions, and how they relate to this work. Section 9 discusses some possible defences of computational functionalism, and their consequences.

Section 10 describes a proposal for how brains could be conscious, without assuming computational functionalism. This proposal centres on the use of an analogue model of 3-D space in the brain. However, other sections of the paper are independent of section 10; if analogue representations are not an answer, the problems raised in the rest of the paper still remain. Section 11 concludes the paper.

## Information in physical devices

This section discusses the nature of information inside physical devices, and the meaning of that information—what it is about. I consider three types of physical device—books, digital computers, and brains. The devices span a huge range of physical complexity and information capacity—from a notebook list of items, to a thousand-page book; from a computer chip in a thermostat, to a cloud-hosted computing estate; from a fly’s brain to a human brain. I address issues which apply across the full complexity range.

In each case, there is a distinction between (i) intrinsic information, which exists physically inside the device, in an encoded form; and (ii) interpreted information, which has meaning about external things and events. To get the interpreted information, you need to decode the intrinsic information, using some separate decoding information. Some types of intrinsic information (I) and uses of decoding information (D) to interpret them, are shown in the table.

The words ‘decode’ and ‘interpret’ are used interchangeably. Generally, decoding information D is used to interpret information of type A **as** information of type B. Several stages of interpretation ‘A as B’ are shown in the table. The lists of interpretations ‘A as B’ are illustrative, and not complete.

In sections 3–5, I describe these cases in more detail—in all cases showing that (i) the decoding information D, required to interpret the intrinsic information I, is external to the physical device; (ii) in the absence of decoding information, the intrinsic information in the device is not about any external thing. It could be about anything, so it is not definitely about anything. It has no defined meaning; and (iii) without any decoded information about external things, the device is not conscious of external things.

In all cases, the intrinsic information I, and the decoding information D, are both created by a process of selection—the selection of designs that fulfil some purpose.

## Information in books

When looking at a book in some unknown script, we do not know what it is about. In the absence of any decoding information, a fragment of the text might be a laundry list, or it might be a description of the year’s harvest, or it could be anything at all. Since it could be about anything, without decoding information it is not definitely about anything.

When you read a book, you consciously experience mental images of what it is about—the things or events that it describes. To experience those mental images, you make the interpretations in Table 1. To do those interpretations, (to decode the book) you use your knowledge of your language and how to read it—information which resides in your head, outside the book.

**Table 1:** Information in physical devices

**Physical device**	**Intrinsic information I, internal to the device**	**Processing in the device (physical changes over time)**	**Decoding information D is used to interpret information of type A as information of type B: ‘A as B’**
**Book**	Ink characters on the pages	Pages are turned by a reader to expose characters	Printed characters as letters Letter sequences as words Word sequences as sentences Sentences as narratives Narratives as meanings, about things and events
**Digital computer**	Currents and voltages in electronic components	Currents and voltages change, following known laws of electromagnetism	Voltages as bits Bit sequences as numbers Numbers as solutions to algebraic equations Algebraic equations as models of physical situations—things and events
**Brain**	Neural spike trains Synaptic connection strengths	Spike trains cause other spike trains and changes to synaptic connections, following complex and partly known laws of biology	Neural firing rates as encoded sense data Firing rates as conditional probabilities of external situations Firing rates as motor commands to muscles (Motor actions as survival and reproduction)

Some of the decoding information D was created by the development of a human language, typically over hundreds of generations. Other decoding information, about reading, writing, and typefaces, is created by people over a few generations. The intrinsic information I in a book is created in days, months, or years by a few people. These are all processes of selection, selecting designs that fulfil their human purposes, and rejecting other designs. Selection creates information, inside and outside the device.

There is an obvious consequence: a book is not conscious of its own meaning. If a book contains the words ‘the *tricoleur* flag fluttered in the breeze’, in itself the book contains no mental image of flags, colours or movement—as you do when you read it. The information inside the book consists only of ink shapes on paper—with no meaning of a moving flag.

In the light of this conclusion, why do some leading theories of consciousness treat the brain as a computer, and as only a computer? The rest of the paper is an investigation of those questions.

## Information in digital computers

A digital computer is a network of electronic components. It works by obeying the laws of electromagnetism, as they apply to those components. This is a complete and accurate description of what the computer does, and it agrees with all the data which we might get by examining it with electrical probes such as voltmeters. We would be surprised if it did not. As this description accounts for all the data we can ever get by examining a computer, in scientific terms, what other description is needed?

We feel that there is more to it than that. Surely it is computing? To say this requires interpretation of the physical events in the computer. Table 1 shows some of the interpretations we need to make, to know that a box of electronics is computing. They form a chain of successive interpretations. At each link of the chain, we could have made a different interpretation:

To interpret voltages as bits, we interpret a small range of voltages as ‘1’, and another range of voltages as ‘0’. We might have used other ranges of voltages, or encoded bits as the relationship between different voltages; or instead of bits, used an N-valued logic with any N.

To interpret bits as numbers, we use a diverse set of encoding schemes such as binary encoding or floating point representations. We could have picked different encodings, or even used some arbitrary mapping from sequences of bits to numbers.

To interpret the operations as numerical operations, there are many choices. Consider two physically identical computers C1 and C2. In C1, we interpret a certain sequence of bits as meaning the number N; in C2, the same sequence of bits is taken to mean 10^N^. The physical operation that we interpret as ‘addition’ in C1 must be interpreted as ‘multiplication’ when we think about C2.

To interpret results of numerical operations as solutions to algebraic equations, there are also many choices. The same number may be the solution to an unlimited set of equations. A computer output of ‘5’ may be interpreted as a solution to the algebraic equation x^2^ = 25. The same output ‘5’, could be interpreted as the solution to x + 3 = 8.

When a computer computes an integral ∫f(x)dx by summing numerical values along a path in the complex x plane, the integral might have taken any other path between the same two end points in the complex plane, and would have given the same result. Or the integral could have been changed by an arbitrary substitution of variables, without changing the numerical result.

We may interpret a set of algebraic equations as representing some physical situation. The same set of equations can represent many different physical situations. Take 12 identical sets of equations of motion (these sets could be x = x_0_ + vt + 1/2at^2^; p = mv; F = ma, E = 1/2mv^2^). These equations might represent the motion of 12 particles in one dimension; or of 6 particles in 2 dimensions; or of 4 particles in 3 dimensions; or some of the equations could represent angular motion.

So there is a chain of many levels of interpretation, to get from physical events in the computer to the external things they might represent. Multiplying the number of choices at each link of the chain, we get an unbounded number of possible ways to interpret the electronic activity in the computer as a computation. A large amount of decoding information D is needed to find the correct interpretation. There is an infinite number of other interpretations—most of which would make no sense to us.

The gist of these examples is that computers are used to solve mathematical problems; and mathematics is so wonderfully flexible and self-consistent, that the same mathematical problem can be solved in an unlimited number of ways, and the same mathematics can represent an unlimited set of different physical situations. A running computer might be doing any of these things. What it is doing is defined only by our interpretation of it; it is not defined solely by events inside the computer.

If a computer C computes some numbers N, as the solution to a set of algebraic equations A, which represent some physical situation P, there is a chain of interpretations 


$$ \mathrm{C}=>\mathrm{N}=>\mathrm{A}=>\mathrm{P}. $$

This is usually shorthand for a longer chain, when the computer has been designed as several layers of ‘virtual machine’. At each link in the chain, there is a large set of possible interpretations we may make. The decoding information needed to make those interpretations is all outside the computer, in our heads and in the heads of computer designers.

If we look only inside a running computer, and have no decoding information to enable us to interpret what it is doing, it might be simulating something, or it might be playing chess, or it might be doing any one of a huge set of other things. Because it might be doing anything, it is not definitely doing any one thing (except following the laws of electronics). This leads to the result that computation is not a physical process. It is an interpretation of a physical process. The words ‘interpretation’ and ‘decoding’ can be used interchangeably.

There are standalone computers, which interact with people; and embedded computers which are parts of some larger physical device. In a standalone computer, the interpretation is done by people. For an embedded computer, interpretation occurs through the physical effects of its outputs, through transducers.

Encryption is encoding, like the encoding of information in a computer. Without knowing a decryption key, an encrypted message is meaningless. Similarly, without decoding, the information in a computer has no meaning.

This can be illustrated by an example. Consider a floor-cleaning robot, which can move, steer, and pick up dust. When it is started in a room, the robot blunders around, bumping into obstacles (which we call A, B, and C). As it does so, it builds up a digital map of the room, so that after a time it can steer around the obstacles. The digital map is encoded in its memory, in a way known to its designer. This encoding knowledge is not accessible inside the robot’s computer.

From the information physically inside the computer, it is impossible to tell which of its memory locations refer to which object A, B, or C—or how that information is encoded. So it is not possible for the computer to be aware of, or know about, object A, based on the information in its memory—because, colloquially, it does not know which bits to look at.

The only way to know about object A is to decode the information in its memory. There are only two ways for this decoding to happen: (i) information about A is decoded by the motor and steering apparatus of the robot, to ensure it does not bump into A; or (ii) if the robot is sent back to its makers for repair, designers may use their knowledge of the decoding to probe the memory locations, looking for faults. Both of these decodings use information outside the computer. Without this decoding, the only process that happens inside the computer is digital electronics. The person-centred metaphors of computers being ‘aware of’, or ‘knowing about’, or ‘having memory of’ obstacles, ‘representing’ things, or even being ‘able’ to do things, have misled us, into mis-construing the physical events inside the robot’s computer, as quasi-mental events.

The decoding barrier is not some mild misty layer which might be penetrated if we look hard enough; it is an absolute barrier. Without external decoding information, physical events inside a computer have no meaning.

Influential papers and books (Bostrom 2003, Chalmers 2022) have argued that we might be living inside a simulation. This conclusion shows that we cannot be inside a simulation. To say that ‘a computer is simulating a world’ is a human interpretation of the electronic processes inside the computer. From the information inside the computer (without decoding information), it might be simulating a world, or simulating anything else, or it might be simulating nothing. There are no simulated things inside a simulating computer. Simulated things exist only as peoples’ interpretations of outputs from the computer. If some beings were running a computer simulation of us, we would not exist inside the computer; we would exist only in their minds. We would be imagined people, just like the imagined people in the books we read.

The problem of interpretation is acute when the computer is used for machine learning and artificial intelligence (Togelius 2024). Then, we need to interpret the bits as representing numbers; the numbers as representing neural activation levels and connection strengths (in a simulated neural net), and the neural net as performing some function—usually seeking some mathematically defined goal.

Without human interpretation, an AI computer is simply doing electronics—nothing else. It might be simulating neurons, or it might be generating random numbers, or it might be doing nothing. There is no intelligence inside the box; all the intelligence lies in the human interpretation of its outputs. This has important consequences for current deployments and discussions of AI, such as (Bostrom 2014, Harari 2020).

As a first consequence, AI is not conscious of external things or events. Information in a computer is not about external things or events (without external information to decode it), so the computer cannot be conscious of them. The only information about things and events is interpreted information, which is in people’s heads, and which depends on decoding knowledge outside the computer. Hence AI is not conscious (Butlin et al. 2023, Togelius 2024).

A second consequence is that some kinds of AI Superintelligence are not possible. When a computer does AI, the intelligence is in peoples’ interpretation of the outputs. IBM’s Deep Blue played brilliant chess because Gary Kasparov interpreted its outputs as brilliant chess. ENIAC printed brilliant log tables because people interpreted them as log tables. If there were no human chess players, Deep Blue could not play brilliant chess; it would just be running electronics. If ‘superintelligent’ computer outputs cannot be interpreted by people (because nobody understands them), there is no superintelligence. This contradicts the many books and papers anticipating superintelligence, such as (Bostrom 2014, Kurzweil 2020).

These two consequences follow for the same underlying reason: the physical events in a computer do not define what it computes. Those events do not define simulated people, or simulated intelligence. People and intelligence are only defined by a person who interprets the physical events as a computation.

The conclusion that AI is not conscious agrees with the No-Go theorem of (Kleiner and Ludwig 2024). This theorem states that if consciousness is dynamically relevant, AI systems cannot be conscious. To be ‘dynamically relevant’ means that consciousness influences the results of AI computations—which it cannot do, as the results of AI computations are fully determined by the laws of physics, applied to electronic components. This paper uses a related, but different argument; that AI computations use only encoded information, which cannot be decoded inside the computer to give meaningful consciousness. The two conclusions agree.

Horsman et al. (2014) give a definition of computation which is more restrictive than the description given here, and their results are consistent with some of the kinds of computing discussed in this paper.

Their definition of computing applies to a computation which has been mathematically specified, and where the specification has been mathematically transformed, to the point where it can be implemented on a physical computer, in a way that is understood by the designers, guaranteeing its conformance with the specification. In the definition of Horsman et al., computers are engineering artefacts, which are understood by their designers. This definition applies to many kinds of rigorously defined computation, but it does not apply to AI computers—where the designers do not understand mathematically how a simulated neural net does what they intended.

In the definition of Horsman et al., and in this paper, encoding of the inputs and decoding of the outputs are essential requirements for a computation to exist. Also essential is a computational entity [which they later called a ‘representational entity’ (Stepney and Kendon 2021)] which is outside the computer, and has the information necessary to encode its inputs, and decode its outputs. For Horsman et al., and in this paper, a computation is a physical process interpreted by a computational entity, which may be a person (but need not be).

Some of the results of Horsman et al. (2014) are anticipated in Cummins (1989), in chapter 8 on interpretational semantics, where he describes the role of interpretation in computation, illustrating it by a ‘Tower Bridge’ diagram similar to the diagrams in Horsman et al.

In later papers, Horsman et al. extend their definition of computing to apply to more diverse kinds of computing—including natural computers in brains. Their conclusions are consistent with the conclusions of this paper—that in all cases, computation is an interpretation of physical events, which depends on an external representational entity to encode information before computation, and to decode it after computation.

That computation is an interpretation of a physical process is clear for older computing devices, such as the abacus—used as a device for adding numbers. What happens in an abacus is not addition; it is the movement of beads. The abacus is designed so that we can interpret these movements as additions; but we could equally interpret them as other things, such as a model of sheep in a meadow, or of fish on the moon, or as predicting the future. The physical events in an abacus do not define a computation. An abacus is only a computer because we interpret it as one.

Some philosophical stances on these issues are discussed in section 8.

## Brains as computers

Animal brains are widely regarded as neural computers. Here I investigate the consequences if the brain is assumed to be a neural computer, and only that**—**if we assume that neural computation (and the things that support it, such as blood flow) are the only events that occur in brains. This is the dominant view today in cognitive science and in medicine. It can be summarised:

Sense data are encoded as neural spike trains going into the brain. Neural spike trains in the brain cause other spike trains in the brain, in a way which we interpret as computation. Spike trains in the brain cause changes in synaptic connection strengths, which we interpret as memory and learning. Some spike trains go out of the brain as motor commands, to be decoded by muscles. Muscular action influences the survival and reproduction of an animal.

As scientists we interpret the brain as a computer, which may for instance compute Bayesian probabilities (Rao et al. 2002, Knill and Pouget 2004, Friston et al. 2006, Friston 2010). We interpret certain neural activity as encoding posterior probabilities, as doing visual edge detection, filtering sounds by frequency, or other functions we understand. These functions are at Marr’s (1982) levels 1 and 2; only Marr’s level 3 (of neural implementation) happens in brains. Marr’s levels 1 and 2 are our interpretation.

Without decoding, there is no meaning inside a computing brain, just as there is no meaning inside a running digital computer, or in a book. There is a quantitative difference between man-made computers, and brains considered as neural computers, concerning the amount of decoding information (called D in section 2) which is required in each case, to interpret physical events in the computer or brain as being about external reality. We expect D to be much larger for brains, than it is for man-made computers:

In man-made computers, the designs are generally kept simple where possible. For most computer applications, the decoding information D, required to interpret physical events inside the computer as being about external things, is large but knowable—typically accessible in many pages of design documentation.

We are only beginning to understand how events in the world are neurally encoded in brains. There is a huge amount we do not yet know. There are thousands of types of neuron in the brain; we only tentatively know how very few of these types encode information. One neuron has thousands of input synapses and thousands of output synapses; we only partially understand how these all encode information. The amount of decoding information D that we do not yet know probably far exceeds the little we know. We should therefore expect the decoding information D for brains to far exceed the decoding information for man-made computers.

Any neural firing must be accompanied by some form of metadata which defines what the firing represents. A single neural firing rate, like a single register in a digital computer, has no defined meaning. The metadata defining its meaning may be static (e.g. defined by the structure of the brain) or may be dynamic. Modern computing practice suggests that dynamic metadata plays an important role. Neural plasticity suggests the same thing in the brain; cortical columns can be re-purposed to play different roles. How is the dynamic metadata in the brain represented?

A possibility is that for every neural data pathway, there are parallel metadata pathways defining what the data represents. Data pathways and metadata pathways have different roles and requirements. A metadata pathway may need to be more information-rich, more widely ramifying (serving many neural data pathways), and more slowly changing, than a data pathway. The relationship between data pathways and metadata pathways in the brain may be far from a simple 1:1 relation.

The presence of dynamic metadata in the brain makes the problem of decoding neural activity more complex. Simple associations like ‘The firing rate of neuron X means A’, that have been found for some neurons may be in the minority. It may be more typical to find: ‘The firing rate of neuron X means A or B or C, depending on the firing of neurons U, V, W…’. If consciousness requires decoded information, this is the kind of complex decoding barrier that neural theories of consciousness would need to overcome.

Brains are embedded computers, whose outputs are motor commands. Before there were any scientists, the only interpretation of brains (by which they are computers) was in muscles from motor commands, and the resulting impact on survival and reproduction. The resulting selective survival is the way brain activity is decoded, and is also the way brains were designed. This selection process has designed the encodings and decodings (sense organs and muscles). Decoding information is external to the brain, and the decoding is through motor actions and survival. Decoding into actions is not the same as the decoding required for conscious experience of external things.

Continuing the work of Horsman et al. (2014), Stepney and Kendon (2021) consider how it applies to biological computers. They describe an example of bacterial motion, in which sensors at one end of a bacterium detect external chemicals, producing a chemical X inside the bacterium; a chemical reaction in the bacterium makes chemical Y from chemical X; and an actuator in the body of the bacterium produces motion in response to chemical Y.

In terms of the computational analysis, the stimulus ‘food nearby’ is encoded as chemical X; the computation is the chemical reaction X = > Y; and the output Y is decoded as motion (sometimes towards a food source).

Thus the computation is an interpretation of the chemical reaction X = > Y, including an encoding of external information to create X, and a decoding of Y into motion. The ‘representational entity’ which does the encoding and decoding is the body of the bacterium—specifically, its sensor and its motor.

The discussion in Stepney and Kendon’s study (2021) can be generalised to any animal brain. The representational entity consists of the animal’s sense organs (which encode external stimuli as neural spike trains) and its muscles (which decode spike trains as actions). Neural events inside the brain are interpreted as computation, through this encoding and decoding. Computation by the brain is an interpretation of physical events in the brain by a representational entity, which is the animal’s body.

This is the basis of a biological account of how brains have evolved and what they do. It is quite similar to Millikan’s (1984, 1989) Teleosemantics, which will be discussed in section 8. But it has missed out one element which we know is present in brains—phenomenal consciousness of meanings, about the external world.

## Consciousness as evidence about the brain

In this section, I consider consciousness not as a scientific or philosophical problem to be solved (Chalmers 1996), but as a source of evidence about how brains work.

Consider two experimental facts: The first is that phenomenal consciousness arises from events in brains. This is an experimental fact, because some kinds of damage to brains interrupt consciousness; and because when sense data about some external thing does not arrive at the brain (for whatever reason) we are usually not conscious of that thing being present.

The second fact is that consciousness includes consciousness about external things and events. This is what consciousness is like (Nagel 1974). Spatial consciousness is about external things located in a consciously experienced space.

Now consider the hypothesis that the brain is a neural computer, and only a neural computer. This hypothesis is not consistent with the first two empirical facts about consciousness.

If the brain is only a computer, the information in the brain about external things is encoded information. Without decoding, that information might be about external things (such as nearby sources of sense data); or it might be about anything at all, or about nothing. As the decoding information is external to the brain, there is no external thing that the information inside the brain is definitely about. Without decoding, the meaning of information in the brain is indeterminate.

There is no information in the brain which is definitely about any single thing, such as a coffee cup. If this is so, your brain cannot be the cause of your consciousness of your coffee cup; causation cannot create information about a coffee cup out of encrypted information. The information needed to decode the events in your brain—to make them into information about a coffee cup—is not available inside your brain, for a law of consciousness to work on.

If the brain is only a computer, the only way for it to realise consciousness would be if some bridging law of consciousness had access to decoding information outside the brain, by some ‘spooky action at a distance’—for the bridging law to somehow pick out decoding information from the environment. This kind of unconstrained non-locality could not be part of a constrained, testable scientific theory. The theory would be too unconstrained—too easy to bend, to fit any data—so it could never be tested or refuted, and it would never become a scientific theory.

The conclusion is: if your brain is a neural computer (and nothing more) it cannot be the source of your consciousness. In order for consciousness to be about anything, your brain must contain something else, as well as a neural computer.

To reach this conclusion, we have not had to say anything positive about what causes consciousness, or to solve Chalmers’s (1996) hard problem of consciousness. We have only made a negative statement—that if the brain is only a computer, it is not the source of consciousness about external things, because the required information is encoded, and is not accessible in brains.

This conclusion follows from a very general analysis of the nature computing, based on the fact that computing uses encoded information. Some people may find the conclusion counter-intuitive. The supplemental material to the paper explores some of the intellectual background of our intuitions about these issues.

## Implications for theories of consciousness

This paper uses our phenomenal consciousness as evidence, to show that brains must contain something else, as well as a neural computer. Here I discuss the implications of this conclusion for theories of consciousness.

The central conclusion of the paper implies that computational sufficiency (Chalmers 2011), or equivalently, computational functionalism (Butlin et al. 2023) (the thesis that ‘performing computations of the right kind is necessary and sufficient for consciousness’) is not correct. That thesis is a ‘mainstream—although disputed—position in philosophy of mind’. Since computational functionalism is the basis of most current theories of consciousness (Butlin et al. 2023), this paper implies that those theories cannot be correct. To defend any purely neural theory of consciousness, you would need to refute the results of this paper.

Most current theories of consciousness assume that the brain does a certain kind of computation C, which includes computational processes P1, P2, P3,..—some of which cause consciousness. Some of the computational processes which are claimed to cause consciousness are:

- P1 is ‘representing objects in the visual field with recurrent processes’ (Crick and Koch 1990, Lamme 2020)
- P2 is ‘making Bayesian maximum likelihood models of reality’ (e.g. Rudrauf et al. 2017, Deane 2021)
- P3 is ‘higher order computation about the brain’s own computations’ (Brown et al. 2019, Lau 2019, 2022)
- P4 is ‘making information globally available in the brain’ (Baars 1997, 1988, Dehaene and Changeux 2011, Dehaene 2014)
- P5 is ‘linking computational units, with high connectivity’ (Tononi 2012, Tononi and Koch 2015)
- P6 is ‘predictive processing’ (Seth and Hohwy 2021, Hohwy 2022)

The problem with these theories is that the computation C is not a property of brains. It is a property of our interpretations of brains as computers. The processes P1… P7 do not physically occur in brains; they happen in our interpretation of brains as computers. What happens in brains can be interpreted as processes P1, P2, and so on; but the same physical events could be equally interpreted as a different set of processes Q1, Q2, Q3… The correct interpretation (P rather than Q) is not defined inside a computer brain, because the physical events in a computer do not define what it computes. So it is not possible for processes like P3 to cause consciousness. Any theory of consciousness which depends on any computational process causing consciousness, cannot be correct as it stands.

We can illustrate the problems by adapting the example in section 4, of the floor-cleaning robot. Suppose we have found the correct interpretation of the brain as a computer. As neuroscientists, we know precisely which neurons in the brain implement the computational process P1 (visual perception), P3 (higher order reflection, or meta-level computation), and other processes. We have gone further, to determine which neurons implement computational processes such as P1A (visual perception of object A) and P3B (higher-order reflection about perception of object B), and so on. All this has been verified experimentally. We then propose a theory of consciousness—that phenomenal consciousness of object B is caused by the computational process P3B. What form might that theory take?

The information in the brain about object B is neurally encoded, and we (as scientists) know how it is encoded; we know which neurons (N1, N2,…) implement the computational process P3B, and how they do it. This is our knowledge, and it is not accessible inside a non-scientist’s brain. So it is not possible to formulate any theory of the form: ‘Information physically inside a brain (including the information in neural events N1, N2, …) causes awareness of object B’. This is not possible, because the information physically inside a brain is not sufficient to define which neurons N1, N2.. implement the process P3B, about object B. That could only be done using extra decoding information, which is not accessible inside the brain in question—it is in the scientific community. Information inside a brain is not sufficient to define the content of consciousness.

It is no more possible for a computer brain to be aware of external things, than it is possible for a floor-cleaning robot to be aware of things. It is impossible in both cases.

Although the proponents of integrated information theory (IIT) (Tononi 2012) have rejected computational functionalism (Findlay et al. 2024), IIT is not consistent with the conclusions of this paper. IIT depends on complexity of computation; it involves a measure Φ of computational complexity, and Φ is required to be large for consciousness to exist. Φ is not an intrinsic physical property of brains. It depends on an external interpretation of physical processes in brains as a computation—a very complex interpretation. This paper shows that more complexity implies more indeterminacy of meaning, which is not consistent with consciousness being about external things. To defend IIT, you would need to refute the conclusions of this paper.

## Philosophical implications of the conclusion

The subject matter of this paper—computational functionalism—has been the focus of much philosophical discussion. This section addresses how that discussion relates to this paper.

I take a pluralist approach to the philosophy of the mind: if two treatments seem to reach parallel, or even antiparallel, conclusions, it may not be the best strategy just to replace one by the other, or to choose between them. Rather than label them with some ‘ism’ and set them in opposition, we need to consider in detail how each one defines the terms in its conclusions, and the arguments it uses. In so doing we may find differences of focus and emphasis between them, and find that each has merit. This sounds rather obvious in general, but may be less obvious in specific cases.

A pluralistic approach to philosophy parallels a pluralistic approach to science—where the same physical system may be described in several different mathematical frameworks, such as Newtonian and Lagrangian dynamics, drawing different insights from different approaches.

The conclusion of this paper is not simply against computational functionalism; it is a specific conclusion, that if the brain is considered as a computer (and as *only* that), certain facts about consciousness cannot be accounted for. The conclusion results from a simple and direct argument, focusing on encoding and decoding in physical computers, and on the indeterminacy of decoding.

With this background, I consider a few of the arguments for and against computational functionalism, including those by (Lucas 1961, Putnam 1988, Penrose 1986, Buechner 2018). There have been many and subtle discussions of this topic, which cannot be summarised here—except possibly to say that the results so far are inconclusive—including both pro- and anti-computational functionalist results.

At a risk of over-simplification, I mention the focus of these discussions. They are not simply about the binary question: ‘Is the brain is a computer’, but are more about the computational **capabilities** of the brain—asking: ‘can brains compute beyond the capabilities of the Church-Turing thesis?’ (Church 1936, Turing 1937), and often appealing to Gödel’s (1931) theorems. Most authors agree that the brain has computational capabilities; and they would agree that these involve the physical encoding and decoding of information. But encoding has not been their focus.

This paper is focused on issues of encoding and decoding; and its simple arguments involve no considerations of higher mathematics. So the conclusions and arguments of this paper are different from those results, and in a sense complementary; they deserve consideration on their own. They are distinct from any debates about higher mathematical capabilities of the brain.

There are proposals for hypercomputation, reviewed in (Copeland 2002), whose concern also is about the extent of computational capability. They aim to extend computational capabilities beyond those implied by the Church–Turing thesis (Church 1936, Turing 1937). Some of them call on physically implausible mechanisms—such as representing real numbers with infinite precision (i.e. with infinite information content) or performing an infinite number of computing steps in a finite time. Extending the computational capabilities of the brain, as in these proposals for hypercomputation, is not a concern of this paper.

The refutations of computational functionalism in (Searle 1980) are well known and have been disputed. One might regard the conclusion in this paper as being just like Searle’s Chinese Room result. However, while this paper reaches the same conclusion as Searle’s, the arguments are different.

Searle’s Chinese Room is an argument by analogy—an analogy linking a person in a sealed room, doing a well-defined task, to some part of a brain, or part of a digital computer. Argument by analogy is not rigorous, because analogy depends on similarities between two domains, and the similarities have no well-defined limits; they are in the eye of the beholder. Searle’s use of analogy in the Chinese Room argument has rightly been questioned.

The conclusion of this paper does not rely on analogy. It relies on evidence about actual computers and brains, and on the fact that all information in brains is encoded. To follow this argument, we need not make any mental segues from sealed rooms to neurons. So while the conclusion of this paper agrees with Searle’s anti-functionalist conclusions, the argument is entirely different. Arguments against Searle are not arguments against the conclusion of this paper.

There is an argument, for instance in Putnam (1988), Searle (1992), Sprevak (2018), that computation is a ‘trivial’ property of any physical system, such as a rock; that there are internal physical processes which can always somehow be mapped onto any computation, leading to pancomputationalism. This issue may be one of definition. If you define ‘computation’ purely in terms of the physical processes inside a computing device, then possibly every physical system computes. However, if, following Horsman et al. (2014), you require an external ‘representational entity’ to encode and decode the computation, and as this paper does, the spectre of pancomputationalism vanishes.

This paper views a computation as not just a set of physical events in a rock or a brain, but as a set of physical events, together with an external interpretation (or decoding) of those events. For instance, when we say: ‘the brain is a Bayesian computer’, this is our interpretation of the brain, that the brain calculates Bayesian probabilities. The Bayesian brain is a successful scientific theory, agreeing with a lot of data (Rao et al. 2002, Knill and Pouget 2004, Friston 2010). We can say no such thing about a rock.

Chalmers (2011) defines ‘computational sufficiency’ (which is equivalent to computational functionalism) as the thesis that ‘the right kind of computational structure suffices for the possession of a mind’. He defines computation in terms of ‘causal organisation’, or ‘causal topology’—the idea that the causal network of a physical computer mirrors the causal network of an abstract computation. He argues that even when a computation is semantically ambiguous in ‘what it is about’, at a syntactic level it is well-defined by a causal topology, which resides entirely inside the computer.

Chalmers’ syntax/semantics distinction is not the same as the distinction made in this paper, between the mathematics being computed, and what the mathematics represents. In this paper, an encoded computation is indeterminate both in the mathematics it computes (as in the example in section 4, of addition versus multiplication), and in the physical situations that the mathematics represents. Chalmers’ syntactic level does not even define the mathematics being computed. So we may question the usefulness of syntactic determination, as it does not determine the mathematics—still less does it determine the physical situation it represents.

We may also question Chalmers’ use of causal topology to define syntactic computation. Is the statement ‘A causes B’ a statement about the conditional probability of A given B, *versus* the probability of A given not B? Does it therefore depend on a counterfactual of B? At the micro level, is the behaviour of a physical computer determined by basic laws of nature, which have time-reversal symmetry (and therefore, may not imply causality)?

These are not straightforward questions. They imply that Chalmer’s definition of computation may depend not just on the physical events inside a computer, but also on some external abstraction, or interpretation, of those events. This could be consistent with the analysis of Horsman et al. (2014), and with the conclusions of this paper—that computation without external interpretation cannot account for consciousness.

There has been extensive philosophical discussion of the related topics of mental representation, intentionality, and semantics. These terms have overlapping meanings, and are used in different ways by different authors. They are related to this paper, because mental representation can be regarded as mental encoding of information, including information about the outside world. The term ‘encoding’ is rarely used in those philosophical writings, but work on representation relates to the work of this paper, and there are insights to be drawn from it.

The work I am referring to has two main starting points—human language, and animal cognition. Sometimes what flows from these is hard to distinguish. Work starting from human language includes (Tarski 1956, Davidson 1967, Fodor 1975, Dretske 1981, Kripke 1982). It typically focuses on truth-conditional semantics—the conditions (or ‘possible worlds’) for a linguistic expression to be true, or to refer to something—and then extrapolates from linguistic expressions to the contents of the mind, as in a Language of Thought (Fodor 1975). In that work, essentially all semantics are truth-conditional semantics.

Cummins (1989, 1996, 2010) has given convincing reasons why language understanding involves more than just language-like symbols in the brain. It must also involve representations—in a sense which he distinguishes from atomic ‘indicators’, in that his representations have structure, which typically matches the structure of their ‘targets’ in the world. The structure of a representation defines its ‘content’, which is to be distinguished from any target. Cummins also addresses non-linguistic uses of representation, and animal cognition.

The considerations of this paper are closer in focus to those of Cummins, than to work on truth-conditional semantics. This is partly because consciousness may be linked more to animal cognitive capabilities, than to anything specifically human, such as language. The converse, that consciousness is essentially and only human, cannot be assumed.

Millikan (1984, 1989) also takes a more biological, evolution-oriented approach in her ‘teleosemantics’—grounding the concept of a representation in its use by ‘consumer’ systems in the brain, in historically ‘normal’ situations. Cummins and Millikan take different approaches, and there are disagreements between them—for instance (Cummins 2010 ch. 8) accuses teleosemantics of a ‘chicken and egg’ evolutionary circularity. But in a pluralistic approach, these differences are not the point. Both Cummins and Millikan have valuable insights into mental representation, and thus into mental encoding—the focus of this paper.

## Defences of computational functionalism

Proponents of computational functionalism, or of theories of consciousness which depend on it, may look for ways to rebut or avoid the conclusions of this paper. I cannot anticipate these objections in detail, but I can describe some available choices, and the consequences of those choices.

The conclusion of the paper is that if the brain is a neural computer, and only that, it cannot be conscious. The argument to the conclusion has the following steps:

1. Consciousness arises from physical events in the brain
2. Consciousness contains information about the external world, which is not encoded information
3. All information in a brain is encoded in patterns of neural firing
4. To decode that information into consciousness, decoding information is required.
5. The decoding information does not reside in the brain.
6. Where does it come from? There is no good way to get it.

To avoid the conclusion, you need to reject one of points (1)–(5), or answer question (6). This section describes some possible ways to do this, and the choices they entail.

Point (1)—that consciousness arises from physical events in the brain—is widely, but not universally, believed. To reject it, you would need both to ignore widespread evidence that damage to the brain interrupts consciousness, and to take some exotic metaphysical stance, saying for instance that consciousness of external things arises from the things themselves. There are views like this—for instance, linked to enactivist views that ‘the world is its own best model’, and claiming that we are somehow conscious of that model. But they require some metaphysical extravagance—including, for instance, a rejection of the locality of classical physics, with nothing to put in its place.

Regarding point (2), it is rarely argued that consciousness does **not** contain information about the world—although there are positions holding that ‘consciousness is an illusion’. In that way, by denying the data about consciousness, you would remove the possibility of doing any science (which, to a scientist, is an unattractive prospect).

We can question whether the information in consciousness is ‘not encoded’, or question what is meant by ‘not encoded’. These are not trivial questions. The meaning of ‘encoded’ in this paper has been largely defined by examples, rather than by verbal definition. It may help to regard encoding as not a binary issue, but as a matter of degree (defining the degree of encoding by ‘how much decoding information is required, in order to decode’). It seems that spatial consciousness—‘what it is like’ to be conscious of space—is very like real space. Straight lines in consciousness are straight lines in real space, and boundaries in consciousness are boundaries in real space. If the information in spatial consciousness is encoded, this may be regarded as a ‘light’ encoding, (requiring little or no decoding information) compared with a ‘heavy’ encoding of space in neural firing rates.

The geometric fidelity of consciousness can be questioned, for instance from the stance that ‘perception is an illusion’; that the world is not as we perceive it to be (e.g. Prakash et al. 2020, 2021); for instance, that what we consciously experience as straight lines are curves or even surfaces in real space. This milder form of illusionism does not remove all possibility of doing science, but it calls into question all the successes of the physical sciences; scientifically, this feels like a backward step.

Point (3)—that all information in a brain is encoded in patterns of neural firing—follows from the conventional neural computer model of the brain. This is a position that neuroscientists are unlikely to disagree with. Some might argue that neural encoding is a ‘light’ form of encoding, and that it is sometimes ‘obvious’ how neural firing rates are used in the brain (what firing rates ‘mean’ or ‘represent’). The reply to this, as in section 5, is that (i) the encoding of 3-D space by neural spike trains in time cannot be regarded as a ‘light’ encoding—it requires large amounts of decoding information; and (ii) what is known to neuroscientists is not information available inside the brain, for a law of consciousness to depend on.

Philosophical treatments of computation have sometimes paid little explicit attention to the question of encoding. But they have not (to my knowledge) denied that physical encoding is an essential part of computing in the brain. In any case, encoding is a brute fact, exemplified by all the forms of computation that we know about, both in brains and digital computers, as in section 4 and 5 of this paper.

Point (4) is that to decode encoded information, you need to know the coding system. That knowledge of the coding system is extra information, which I have called ‘decoding information’. This is a commonplace of all systems of encryption—where, without knowing the key, an encrypted message conveys no useful information and has no meaning. It seems hard to avoid this point.

Point (5) is that the information required for decoding (the decryption key) does not reside inside the brain. In the analysis of Horsman et al. (2014) that information is outside the brain, in an external ‘representational entity’. A possible counter to this is that the brain may contain some higher order meta-computation about its own computation, as in (Lau 2019, 2022); and that the meta-computation contains information about how the base level computation is encoded. This argument fails simply. Because the meta-computation is itself encoded, any decoding keys are encoded, and are inaccessible inside the brain. Decoding by meta-computation is prey to a simple infinite regress. I know of no workable answers to this.

Finally, is there a good answer to the question (6)? If decoding information is not available inside the brain, where does it come from? There are possible answers. For instance, laws of nature have information content. Perhaps there is a Bridging Law for consciousness (Chalmers 1996); a law of nature, which is the source of the decoding information. That answer has two unattractive features. First, the neural encoding in the brain is a heavy encoding, requiring large amounts of decoding information. In contrast, the known laws of nature ‘can be written on a T-shirt’—that is, they have very small information content. This is for good reason. If a law of nature had very large information content (i.e. if it had many variable parameters), it would be impossible to test or refute, because its parameters could be adjusted to fit any possible data. In the Bayesian Philosophy of Science (Sprenger and Hartmann 2019), an untestable hypothesis is not a candidate for a theory. The law of consciousness cannot have a large enough information content to decode the neural firing in the brain, and still be testable. Second, even if the law was somehow allowed to have large information content, such a law of nature would seem to be parochial and unattractive—precisely adjusted to work for only one species on one planet. I know of no workable solutions to these problems; solutions may exist, with some metaphysical costs.

I suggest that these are some of the options for avoiding the conclusion of this paper, and these are their costs. Some may be prepared to pay the costs. An alternative, which may be simpler, is to relinquish computational functionalism and explore the alternatives in the next section.

## Analogue models of reality

If computers alone cannot be the source of consciousness, what else might be happening in brains, which could be the source of consciousness? Brains may contain an analogue model of local space; and an analogue model contains information about reality that requires little or no decoding. This could be the source of spatial consciousness. However, I emphasise that the idea of analogue representations is mentioned just as an avenue for possible investigation, not as the unique solution to the problems raised in this paper. There are arguments that analogue representations require some decoding, and do not solve the problem of decoding in computers. If they do not solve the problems raised in this paper, that does not alter the main conclusion of the paper—that there are such problems.

What is an analogue model? We can approach this question by examples:

- A toy car is an analogue model of a real car
- A paper map is an analogue model of an area of territory
- An image from a convex lens is an analogue model of a scene
- A photograph on film is an analogue model of a scene
- A reconstructed hologram is an analogue model of a scene

Each analogue model has a point-for-point structural correspondence with the thing it models. This means that if two points are neighbours (limitingly close to each other) in the thing being modelled, the points in the model that model them are neighbours.

To define what an analogue model is, the relevant branch of mathematics is topology. For example, consider a physical system P, which is the surface of a solid object. The analogue model M of P is an image of the object (possibly distorted) produced by a lens.

Topology is relevant because M and P have the same topology. If P is a doughnut, M must be doughnut-shaped. If P consists of two separate objects, M must contain two separate shapes; and so on. There is a mapping function f between P and M. If m is a point in M, and p is a point in P, then m = f(p). f preserves locality and neighbourhoods; it is a continuous function. If two points in P are neighbours, the corresponding two points in M are neighbours.

For instance, if P is coloured in three different colours A, B, and C, then M must be divided into regions with three properties D, E, and F, with a single mapping between {A, B, C} and {D, E, F}. Junctions of regions must correspond between M and P. If a point p in P is in the junction of regions coloured A and B, the corresponding point m in M (the point m related by m = f(p)) must be in the junction of regions in M with properties D and E. To support these properties, the mapping function must be a fibre bundle.

From these considerations, the model M contains information about P (for instance, information about the junctions between regions) which requires no decoding.

These analogue models have a spatial nature, which may be in a space of any dimensionality. That is needed to support the continuity property, described above.

I call this property spatial binding. Spatial binding is a property of an analogue model. It holds in the examples above. It means that some kinds of information which are true in reality (such as the existence of a border between regions of two different colours) are also true in the model, with no need to decode the information in the model.

That is the fundamental difference between an analogue model and an encoded model, as used in a computer. Some information in an analogue model of reality is not encoded, and there is no need to decode it. There is no decoding barrier.

Our consciousness includes consciousness of things, distributed in a consciously experienced space. The concept of a ‘thing’ is loose and informal; perhaps a thing is an association of properties (such as redness and roundness) in the same spatial neighbourhood. We would like spatial consciousness also to apply to other cases—such as an irregular surface of mud or rock—where discrete ‘things’ are not distinguished.

These cases are covered if we say that consciousness has spatial binding—the ability to discern different properties (such as colour, hardness, sound, or inertia) at the same spatial location, or in neighbouring locations.

Empirically, consciousness has spatial binding. We experience a spatial consciousness, which is like a three-dimensional model of local space, populated by properties at locations in the space. The conscious model has a continuity property, in that two points which are neighbours in real space are also neighbours in conscious experienced space. So consciousness includes an analogue model of local physical space.

This raises the possibility that consciousness arises from an analogue model of space, which exists physically in the brain.

That offers a possible way through the previous *impasse*—that consciousness cannot arise from a brain which is only a computer. In this proposal, the brain also contains an analogue model of reality, which requires little or no decoding to give consciousness of reality. Both the analogue model and consciousness have spatial binding.

A possible objection to analogue models is that if you look closely at an analogue model, it is divisible into parts which are encoded; so it needs some decoding. For instance, a map consists of marks on paper, encoded in ink; a photograph is encoded in dye pigments; a digital map consists of encoded pixels; so some decoding is needed. This has been used to argue that analogue models and digital models cannot be distinguished.

This objection applies to some analogue models, not all. An image from a lens is not infinitely divisible; a hologram (being a Fourier transform of a spatial distribution) is not infinitely divisible. In both cases, the minimum length of divisibility is the wavelength of light. A single quantum state is not divisible, and some quantum states can hold macroscopic amounts of information, for times much longer than typical quantum decoherence times of 10^−18^ s (Tegmark 1999). These long-lived states are Bose–Einstein condensates (BECs), such as superconductors or superfluids (Bose 1924, Einstein 1925, Pitaevskii and Stringari 2003).

BECs are a possible substrate for an analogue model of space. As a possible substrate for consciousness, they have some attractions: (Lockwood 1989, Cairns-Smith 1996, Worden 1999):

- As a single quantum state, a BEC is indivisible. This correlates with the observed unity of consciousness.
- A single BEC can hold macroscopic amounts of information, which matches with the large amount of information we have at any moment in spatial consciousness.
- A BEC state can hold information for unlimited times, which is not subject to rapid decoherence (Tegmark 1999). This makes a BEC suitable to serve as a short-term spatial memory in the brain, at least for the sub-second timescales over which conscious awareness varies.
- BECs are a very rare state of matter. Linking consciousness to BECs would avoid a taint of panpsychism.

The key requirement for an analogue model of space is that some of the information in an analogue model—not necessarily all the information—is not encoded, or requires very little decoding information to decode it.

In the philosophical treatment of analogue models in the brain, the work of Millikan (1984, 1989), Cummins (1989, 1996, 2010), Camp (2018), Block (2023) may be particularly relevant. When Cummins illustrates his concept of representation (in contrast to his symbolic ‘indicators’), he emphasises the internal structure of a representation, which defines its ‘portable’ content. He repeatedly uses the examples of maps and models, which are at least steps along the way to an analogue representation.

Is there such a thing as a fully analogue representation, or are all representations partly encoded? The examples I have given (particularly optical images, holograms and BECs) suggest that there is some way to go, in analysing the possible biophysics of analogue representations in the brain, before we can properly pose the philosophical questions. For instance, BECs are quantum mechanical phenomena. Quantum mechanics challenges many philosophical arguments and intuitions.

The question of possible analogue models in brains is a complex one, and is not resolved. If there is analogue model of external reality in the brain, it raises many questions:

- What is it a model of, and how does the brain maintain it?
- What is the biophysical substrate of the model?
- What is the biological use of such a model in the brain? How does it increase fitness?
- What is its impact on cognitive modelling of the brain?
- Does an analogue model in the brain give a possible account of phenomenal consciousness?

These are large questions, and are not addressed in this paper. Some of them are addressed in Worden (2024), and will be the subject of further work.

## Conclusions

The main conclusion of this paper is a negative one—that a computer cannot be conscious of external things. This is because information inside the computer is physically encoded, and cannot be decoded inside the computer to provide the information content of consciousness.

Negative results in science can have far-reaching implications. They show what is not possible, and clear the ground for new ideas. The implications of this conclusion for cognitive science could be as far-reaching as the implications of Godel’s Incompleteness Theorems for mathematics (Gödel 1931).

The conclusion has important consequences for our understanding of man-made computers. Because the information inside them is encoded, it is misleading to think of them with the anthropomorphic metaphors of ‘intelligence’, ‘awareness’, and so on, which we have habitually used. We need to re-set our intuitions about computer applications, including simulations and AI.

The conclusion has major implications for theories of phenomenal consciousness. It implies that the ‘brain as a computer’ interpretation, used in many current theories, does not account for consciousness. In section 10, I outlined an alternative proposal, that an analogue model of reality in the brain is the source of phenomenal consciousness.
